# The Impact of COVID-19 on Admissions and Management of Patients with Atrial Fibrillation Episodes in the Emergency Department

**DOI:** 10.3390/ijerph18116048

**Published:** 2021-06-04

**Authors:** Łukasz Bilaszewski, Wojciech Timler, Katarzyna Budrewicz, Michał Marczak, Remigiusz Kozłowski, Joanna Wizowska, Małgorzata Timler, Dariusz Jagielski, Michał Dudek, Paweł Rasmus, Dorota Zyśko, Dariusz Timler

**Affiliations:** 1 Department of Emergency Medicine, Wroclaw Medical University, 50-556 Wroclaw, Poland; lukasz.bilaszewski@umed.wroc.pl (Ł.B.); katarzyna.budrewicz@umed.wroc.pl (K.B.); joanna.wizowska@umed.wroc.pl (J.W.); dorota.zysko@umed.wroc.pl (D.Z.); 2 Department of Emergency Medicine and Disaster Medicine, Medical University of Lodz, 92-212 Lodz, Poland; wojciech.timler@stud.umed.lodz.pl; 3 Department of Management and Logistics in Healthcare, Medical University of Lodz, 90-131 Lodz, Poland; michal.marczak@umed.lodz.pl (M.M.); malgorzata.timler@stud.umed.lodz.pl (M.T.); 4 Center of Security Technologies in Logistics, Faculty of Management, University of Lodz, 90-237 Lodz, Poland; remigiusz.kozlowski@wz.uni.lodz.pl; 5 Centre for Heart Diseases, Department of Cardiology, 4th Military Hospital, 50-981 Wroclaw, Poland; dariuszjagielski@gmail.com; 6 Department of Anaesthesiology and Intensive Therapy, Medical University of Lodz, 90-153 Lodz, Poland; michal.dudek@stud.umed.lodz.pl; 7 Department of Medical Psychology, Medical University of Lodz, 90-131 Lodz, Poland; pawel.rasmus@umed.lodz.pl

**Keywords:** atrial fibrillation, cardioversion, emergency department, COVID-19, pandemic

## Abstract

Background: During the COVID-19 pandemic, the number of admissions to the emergency department (ED) due to a primary diagnosis of atrial fibrillation (AF) has decreased when compared to pre-pandemic times. The principal aim of the study was to assess the frequency of SARS-CoV-2 infections and sinus rhythm restoration among patients who arrived at the ED with AF. Secondary aims included determining whether patients arriving at the ED principally due to AF delayed their presentations and whether the frequency of successful cardioversion for AF was decreased during the pandemic period. Materials and Methods: A retrospective analysis of medical records of patients admitted to two hospital EDs due to AF during July–December 2019 (pre-pandemic period) versus July–December 2020 (pandemic period) was performed. Results: During the study periods, 601 ED visits by 497 patients were made due to the primary diagnosis of AF. The patients were aged 71.2+/−13.5 years and 51.3% were male. The duration of an AF episode before the ED admission was 10 h (4.5–30 h) during the pandemic period vs. 5 h (3–24 h) during the non-pandemic period (*p* = 0.001). A shorter duration of the AF episode before ED admission was associated with the successful restoration of the sinus rhythm. During the pandemic period, among patients with short-lasting AF who were not treated with Phenazolinum, the restoration of the sinus rhythm was more frequent in the Copernicus Memorial Hospital than in the University Hospital (*p* = 0.026). A positive SARS-CoV-2 test was found in 5 (1%) patients, while 2 other patients (0.5%) had a prior diagnosis of COVID-19 disease noted in their medical history. Conclusions: 1. The number of AF episodes treated in these two EDs was lower during the pandemic than non-pandemic period. 2. The patients with AF appeared at the ED later after AF onset in the pandemic period. 3. Successful cardioversion of atrial fibrillation was more frequent during the pre-pandemic period in one of the two hospitals. 4. A difference of approaches to the treatment of short-lasting AF episodes between EDs during the pandemic period may exist between these two EDs. 5. The patients with SARS-CoV-2 infection during the second wave of the COVID-19 pandemic constituted a small percentage of the patients admitted to EDs due to an AF episode.

## 1. Background

The global death toll due to the pandemic of the coronavirus disease 2019 (COVID-19) soon exceeds 3.3 million persons [1]. Most COVID-19 disease deaths are caused by severe acute respiratory infections by the coronavirus known as SARS-CoV-2 [2]. Nosocomial SARS-CoV-2 infection among hospitalized patients is common [3]. Patients’ awareness of this threat could make individuals reluctant to come to hospitals, not only for diagnostic tests but also for treatment [4]. 

Atrial fibrillation (AF) is the most common arrhythmia treated in an Emergency Department (ED), and prompt treatment can make successful cardioversion more likely. AF occurs in up to 10% of emergency department admissions and is the primary diagnosis for 1% of patients admitted to the ED [5]. The rate of hospital admissions for patients with a primary diagnosis of AF was 27.8% in the Blitz-AF study. This rate exceeded the overall hospital admission rate observed among all patients who presented to the ED for treatment [6]. 

AF may occur in up to 7.5% of COVID-19 patients [7]. Contrary to an expectation that the incidence of AF would increase during the pandemic, Schnaubelt et al. observed fewer visits by patients with paroxysmal and persistent atrial fibrillation during the pandemic period than during the corresponding months of the years before the pandemic [8]. 

Provisional recommendations for the treatment of patients with AF include pharmacological cardioversion, avoidance of electro-cardioversion, and the use of transoesophageal echocardiography [9]. During the current pandemic, less is known about the effectiveness of therapeutic management in patients admitted to the ED with a diagnosis of AF. 

The principal aim of the study was to assess the frequency of SARS-CoV-2 infection and sinus rhythm restoration among patients admitted to an ED due to AF. The secondary aim was to evaluate for possible delays of seeking treatment by patients with recent-onset AF and whether such delay, if present, was associated with a lower frequency of rapid and successful cardioversion.

## 2. Methods

This is a retrospective analysis of medical records of the patients admitted to the ED of two hospitals, during two corresponding periods: The “non-pandemic period” (NPP), from July through December 2019, versus the “pandemic period” (PP), from July through December 2020. 

The ED at the University Hospital in Wrocław is one of four EDs in Wroclaw and is the Regional Trauma Center. Wroclaw is a principal city of the Lower Silesia voivodeship and has approximately 3,000,000 inhabitants. Its ED admission rate was approximately 3000 per month, with an average hospital inpatient admission rate of 25%. A total of 28% of its ED admissions arrived via emergency medical services (EMS). The 30-day mortality was 3.3% [10]. The ED has a “catchment area” of approximately 200,000 inhabitants. 

The ED at the Copernicus Memorial Hospital in Lodz is the Regional Trauma Center for the Lodz voivodeship. Lodz is the principal city of the Lodz voivodeship and has approximately 680,000 inhabitants. The Copernicus Memorial Hospital is the principal hospital for Lodz. Its ED admission rate was approximately 2000 per month, with an average hospital inpatient admission rate of 14%. The 30-day mortality was 3.2%. 

### 2.1. COVID-19 Dynamics and Medical Care Structure during Pandemic

The COVID-19 infection was first detected in Poland on 4 March, 2019 [11]. As of December 31st, the total of confirmed cases of 2020 in Lower Silesia was 88,176, and the total of confirmed cases in the Lodz voivodeship was 84 760 [12,13]. The population on 30 June 2020 were 2,898,500 in the Lower Silesia voivodeship and 2,448,713 in the Lodz voivodeship [14,15].

At the beginning of the pandemic, COVID-19 patients were preferentially routed to Infectious Disease Hospitals [16,17]. Beginning in September 2020, this routing was stopped, and most patients requiring inpatient care were admitted to the nearest hospital [18]. With time, each hospital was ordered to make inpatient beds available for COVID-19 patients. Of these, only those patients requiring tertiary care procedures were to be referred to the designated and more extensively resourced “COVID hospitals”.

The ICD-10 code I48 was used to identify patients diagnosed with atrial fibrillation as a cause of ED admission. The study group included only patients with paroxysmal or persistent AF in whom the AF diagnosis was the primary one, as verified by the review of each subject’s medical record. 

The following data were collected for each patient: age, sex, estimated time since AF onset, treatments administered in the ED, the result of the swab test for SARS-CoV-2 infection, whether there was a history of prior COVID-19 disease, and whether there was a restoration of sinus rhythm in the ED. If the duration of the AF episode was recorded as “unknown” or if it was non-recorded, it was designed as “unknown”.

### 2.2. Statistical Analysis

Data are presented as mean ± standard deviation (SD) for normally-distributed data and as the median and interquartile range (IQR) for ordinal or non-normally-distributed data. The Student‘s t-test and the Mann–Whitney U test were used for statistical inference from these data. Statistical inference from nominal data expressed as rates or frequencies were compared with the Chi-square test. 

Classification and regression trees (CART) analyses were performed in both study periods (PP and NPP) to explore for possible associations in both EDs between sinus rhythm restoration at the ED and:

- patient age (years) dichotomized as ≥65 years or <65 years;
- duration since AF episode onset trichotomized as up to 6 h, 7–43 h, or longer than 43 h (or unknown, as appropriate);
- sex.

Global Cross 102 Validation (CV) cost and its standard deviation were calculated. *p* less than 0.05 was regarded as significant.

## 3. Results

### 3.1. Studied Population

The study group consisted of 497 patients aged 71.2 ± 13.5 years (range 21–97). 255 (51.3%) were male. A total of 310 patients were treated at the University Hospital in Wrocław, and 187 patients were treated in the Copernicus Memorial Hospital in Lodz. The total number of ED visits due to AF was 601, with 389 visits to the University Hospital and 212 visits to the Copernicus Memorial Hospital. 

Between July and December 2019, there were 18,937 admissions to the University Hospital. Between July and December 2020, there were 16,435 admissions. At the Copernicus Memorial Hospital, the numbers of ED admissions were 11,713 and 9221, respectively. 

At the University Hospital, there were 232 AF episodes in 193 patients treated in 2019 and 157 AF episodes in 128 patients treated in 2020. These constituted 59.6% of the episodes of evaluations in the ED for AF in 2020 and 40.4% of all episodes of AF in 2019 (*p* < 0.001). At the Copernicus Memorial Hospital, there were 117 AF episodes among 102 patients treated in 2019 and 95 AF episodes in 85 patients treated in 2020. These constituted 55.2% and 44.8% of all assessed AF episodes, respectively, at that hospital (*p* = 0.032). These data, along with SARS-CoV-2 data, are also presented in Table 1, Table 2 and Table 3. The sum of the number of patients in 2019 and 2020 does not equal the total number of patients across both years because some patients were admitted to an ED both in 2019 and 2020. 

### 3.2. Demographic Data

The sex and age distribution in the pandemic period and the non-pandemic period are presented in Table 1. These did not differ significantly between the two EDs. However, in the Copernicus Memorial Hospital, the patients were older. 

#### 3.2.1. The time since the AF onset to the ED admission

Overall, the mean (and IQR) of the time since the AF onset until ED admission was 10h (4–48 h) during the pandemic period and 5.5 h (3–23 h) in the non-pandemic period, which was significantly shorter (*p* = 0.019). This shorter time during the NPP between AF onset and ED admission was also individually observed at both EDs. 

#### 3.2.2. Restoration of sinus rhythm

Successful restoration of the sinus rhythm in the ED in the University Hospital was greater during the non-pandemic period. However, at the Copernicus Memorial Hospital ED, there was no significant difference between time periods.

#### 3.3.3. Previous and current SARS-CoV-2 infection

A total of 5 patients had positive swab tests for SARS-CoV-2 infection, while 2 patients had a prior history of SARS-CoV-2 infection. The patients with new or prior SARS-CoV-2 infection constituted less than 2% of all studied patients.

#### 3.3.4. The sinus rhythm restoration

The restoration of the sinus rhythm occurred more frequently at the University Hospital during the non-pandemic period, whereas, at the Copernicus Memorial Hospital, there was no significant difference between the periods.

#### 3.3.5. Multivariate analysis of AF restoration

**Figure 1:**
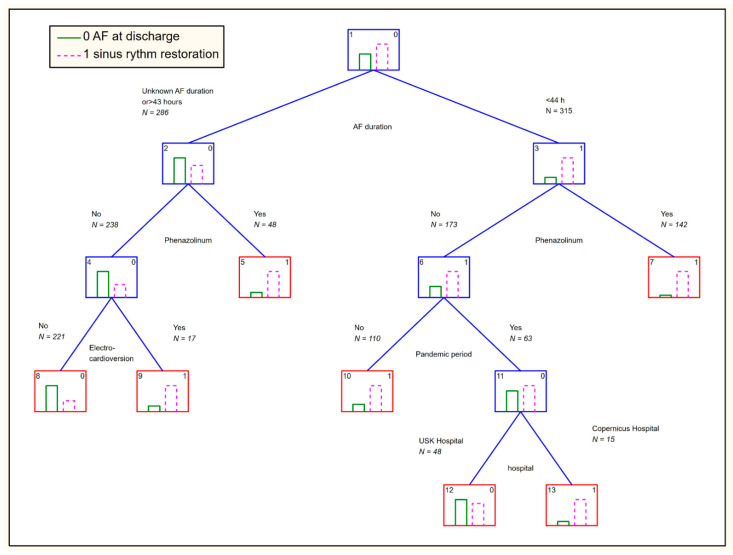
The CART analysis. The dependent variable is the restoration of the sinus rhythm. The independent variables include sex (male or female); age <65 years or ≥65 years; AF duration since onset of 0–43 h vs. >43 h or unknown; AF duration 0–6 h vs. >6 h or unknown; University Hospital vs. Copernicus Memorial Hospital; pandemic period vs. non-pandemic period; electrical cardioversion versus chemical cardioversion using Phenazolinum, Amiodarone, Propafenone, or beta blockers.

The CART analysis for sinus rhythm restoration is presented in Figure 1. Global CV cost = 0.26; s.d. CV cost = 0.019.

#### 3.3.6. CART Analysis

The CART analysis revealed that the most important factor predicting a restoration of the sinus rhythm in the ED is the time interval between AF onset and admission to the ED. A shorter time is more favorable. The treatment of patients during the pandemic era was associated with a lower rate of restoration of the sinus rhythm at the University Hospital, but not at the Copernicus Memorial Hospital. 

## 4. Discussion

We were not able to address the principal aim of the study. The presence of a current or recent SARS-CoV-2 infection was very low in the cohort studied, making any statistical inferences about the restoration of the sinus rhythm in patients with versus without current or recent SARS-CoV-2 infection impossible. Further, the percentage of the patients who attended the ED with a primary diagnosis of AF was approximately 1% during both periods. This finding is consistent with the reported incidence of primary AF diagnosis in a large observational study [6], and it supports the accuracy of the obtained results but illustrates the difficulty of a study of this matter as a single-site study. 

Regarding the secondary aims, there was clear statistical evidence that patients with AF delayed their presentations to the ED during the pandemic period when compared to the non-pandemic period. This may have been related to reluctance on the part of the AF patients to attend the ED because of fear of nosocomial COVID-19 disease. 

This delay in presentation for AF during the pandemic period was associated with a decreased rate of success of chemical or electrical cardioversion back to sinus rhythm at one but not both of the hospitals in which ED patients were studied. This finding might have many causes. Patients with a greater propensity for restoration of spontaneous sinus rhythm may have had a spontaneous return to sinus rhythm at home, and, thus, they did not choose to visit the ED. The tactic of delaying the reporting to the ED in the case of an attack of hemodynamically stable atrial fibrillation represents a new therapeutic option [19,20]. This option was tested in the Rate Control versus 193 Electrical Cardioversion Trial 7-Acute Cardioversion versus Wait and See (RACE 7 194 ACWAS) [21]. This underused, usual option could allow a rapid and spontaneous resolution of many AF episodes. This option may have been unconsciously chosen by AF patients due to fear of nosocomial COVID-19 disease. If this occurred, the consequence could have been that attendance to the ED was done by patients with AF who are more likely to be resistant to treatment, with a lower rate of the restoration of the sinus rhythm rate in the ED. 

Attendance at the ED was less during the pandemic period than during the non-pandemic period. This is concordant with reports of the other authors and with data derived from the ED of the University Hospital from the early phase of the pandemic [4]. The total number of patients admitted to the ED at both the University Hospital and the Copernicus Memorial Hospital was lower in 2020 than in 2019, which is in line with the findings of other authors [8,22].

The management of patients with AF episodes differed between the two EDs. This finding is consistent with other reports indicating significant variation in the emergency management of acute atrial fibrillation [23]. 

## 5. Conclusions

1. The number of AF episodes treated in the ED was lower during the pandemic than during the non-pandemic period.

2. During the pandemic period, the patients with AF arrived later at the ED, and they were less likely to be cardioverted back to sinus rhythm.

3. Differences in approaches to the treatment of recent-onset episodes of AF between EDs during the pandemic period appeared to exist. 

4. Patients with a current or prior SARS-CoV-2 infection during the second wave of the COVID-19 pandemic constituted a small percentage of the patients admitted to the ED because of an AF episode.

## Figures and Tables

**Table 1:** Patients with AF as the primary diagnosis in the non-pandemic period (NPP) and the pandemic period. (PP). Further, the number of COVID-19 positive and convalescent COVID-19 patients are presented.

	University Hospital			Copernicus Memorial Hospital		
	Non-Pandemic Period	Pandemic Period	SARS-CoV-2 Positive /Convalescent in the Pandemic Period	Non-Pandemic Period	Pandemic Period	SARS-CoV-2 Positive /Convalescents in the Pandemic Period
Number of Patients	193	128	3 (1 SARS-CoV-2 positive, 2 convalescents)	102	85	4 SARS-COV-2 positive
Number of AF visits July–December	232	157	3	117	95	4
Number of visits per a patient	1.2	1.2	1	1.1	1.2	1
Age years+/−SD	70.0+/−13.9	70.0+/−15.0	60.7+/−11.1	73.8+/−12.3 *	73.3+/−11.1 *	77.0+/−9.8
Male gender n (%)	107 (55.4)	86 (44.6)	2 (66.7)	63 (49.2)	39 (45.8)	3 (75)

* *p* < 0.05 vs. University Hospital in the corresponding period.

**Table 2:** The distribution of the AF episodes, time since AF onset to the ED admission, the duration of ED stays, and sinus rhythm restoration in the studied hospitals during non-pandemic and pandemic periods.

	University Hospital			Copernicus Memorial Hospital		
	Non-Pandemic Period	Pandemic Period	SARS-CoV-2 Positive /Convalescents in the Pandemic Period	Non-Pandemic Period	Pandemic Period	SARS-CoV-2 Positive /Convalescents in the Pandemic Period
July n (%)	37 (16.0)	29 (18.5)	0/0	23 (19.7)	15 (15.8)	0
August n (%)	36 (15.5)	27 (17.2)	0/0	18 (15.4)	20 (21.1)	0
September n (%)	33 (14.2)	25 (15.9)	0/0	14 (12.0)	20 (21.1)	0
October n (%)	47 (20.3)	26 (16.6)	1/0	21 (18.0)	15 (15.8)	3
November n (%)	34 (14.7)	13 (8.3)	0/0	19 (16.2)	11 (11.6)	1
December n (%)	45 (19.4)	37 (23.6)	0/2	22 (18.8)	14 (14.8)	0
Total July–December n (%)	232 (100)	157 (100)	1/2	117 (100)	95 (100)	4/0
Time since AF onset (h; N*)	5 (3–15.5)	10 (4–48) @	5	6 (2–24)	12 (6–72) @	1
	N* = 164	N* = 111	N* = 1	N* = 70	N* = 61	N* = 1
ED stay duration (min)	362	452 ^	227	322	312	1007
	(227–507)	(290–598)	(243–967)	(88–519)	(180–527)	(550–1162)
Sinus rhythm restoration n (%)	147 (63.4)	78 (49.7) $	2 (66.7)	69 (72.6)	76 (65) #	2 (50.0)

N*—the number of patients with available data. # *p* < 0.05 vs. corresponding period in the University Hospital. $ *p* = 0.007 vs. non-pandemic period. @ *p* < 0.001 vs. non-pandemic period. ^ *p* < 0.05 vs. non-pandemic period.

**Table 3:** The management of AF episodes in the studied EDs during non-pandemic and pandemic periods.

	University Hospital		Copernicus Memorial Hospital	
	Non-Pandemic Period	Pandemic Period	Non-Pandemic Period	Pandemic Period
Electro-cardioversion N (%)	14 (6)	16 (10.2)		1
			23 (19.7) *	8 (19.0)
Phenazolinum n (%)	72 (31.6)	118 (30.3)	37 (31.6)	35 (36.8)
Amiodarone n (%)	14 (6.0)	13 (8.3)	9 (7.7)	15 (15.8)
Beta blocker n (%)	19 (8.2)	23 (14.7)	61 (52.1) *	69 (72.6) *
Propafenone n (%)	13 (3.3)	6 (3.8)	24 (11.3) *	7 (7.4) *

* p < 0.001 vs. University Hospital in period.

## Data Availability

The data presented in this study are available on request from the corresponding author.

## Correction Statement

The Editorial Office and Editorial Board would like to inform readers of a post-publication review process that was conducted related to this publication. Following concerns about a potential conflict of interest between the authors and the original Academic Editor, the Editorial Office has conducted a post-publication peer-review of this article. This process included the recruitment of a new Academic Editor to ensure full compliance with MDPI’s Editorial Process (https://www.mdpi.com/editorial_process). As a result of this process, the original Academic Editor was removed, and a new Academic Editor is listed. The new Academic Editor is satisfied that the editorial process for this article has been completed in accordance with MDPI’s Editorial Process policy.

## References

[1] WHO Coronavirus (COVID-19) Dashboard. https://covid19.who.int.

[2] Zhou P., Yang X.-L., Wang X.-G., Hu B., Zhang L., Zhang W., Si H.-R., Zhu Y., Li B., Huang C.-L. (2020). A Pneumonia Outbreak Associated with a New Coronavirus of Probable Bat Origin. Nature.

[3] Rhee C., Baker M., Vaidya V., Tucker R., Resnick A., Morris C.A., Klompas M. (2020). CDC Prevention Epicenters Program. JAMA Netw. Open.

[4] Chourasia G., Sycz W.K., Wolniakowski I., Dudek K., Porębska B., Moczarska J., Budrewicz K., Wizowska J., Nadolny K., Soko-łowski J. (2020). Changes in the Visits to Emergency Department of Non-Infectious Hospital during the Early COVID-19 State of Epidemic Emerg. Med. Serv..

[5] Russo V., Navarin S., Zampini G., Magrini L., Mann C., Muiesan M.L., De Caterina R., Yılmaz M.B., Beton O., Monzani V. (2013). Management of Atrial Fibrillation in the Emergency Department: Current Approach and Future Expectations. Eur. Rev. Med. Pharmacol. Sci..

[6] Gulizia M.M., Cemin R., Colivicchi F., De Luca L., Di Lenarda A., Boriani G., Di Pasquale G., Nardi F., Scherillo M., Lucci D. (2021). BLITZ-AF Investigators. Europace.

[7] Pardo Sanz A., Salido Tahoces L., Ortega Pérez R., González Ferrer E., Sánchez Recalde Á., Zamorano Gómez J.L. (2021). New-Onset Atrial Fibrillation during COVID-19 Infection Predicts Poor Prognosis. Cardiol. J..

[8] Schnaubelt S., Domanovits H., Niederdoeckl J., Schuetz N., Cacioppo F., Oppenauer J., Spiel A.O., Laggner A.N. (2020). The Impact of the COVID-19 Pandemic on Incidences of Atrial Fibrillation and Electrical Cardioversion at a Tertiary Care Emergency Depart-Ment: An Inter- and Intra-Year Analysis. Front. Med..

[9] Hu Y.-F., Cheng W.-H., Hung Y., Lin W.-Y., Chao T.-F., Liao J.-N., Lin Y.-J., Lin W.-S., Chen Y.-J., Chen S.-A. (2020). Management of Atrial Fibrillation in COVID-19 Pandemic. Circ. J..

[10] Budrewicz K., Dudek K., Porębska B., Wolniakowski I., Nadolny K., Zyśko D. (2019). Short-Term Prognosis in Patients Visiting Emer-Gency Departments. Na Ratunek.

[11] Pinkas J., Jankowski M., Szumowski Ł., Lusawa A., Zgliczyński W.S., Raciborski F., Wierzba W., Gujski M. (2020). Public Health Interventions to Mitigate Early Spread of SARS-CoV-2 in Poland. Med. Sci. Monit..

[12] Koronawirus We Wrocławiu, Na Dolnym Śląsku, w Polsce [RAPORT 31.12]. https://wroclife.pl/nasze-miasto/koronawirus-wroclaw-2020/koronawirus-dolny-slask-31-grudnia/.

[13] Koronawirus w Polsce: Ile zakażeń Dzisiaj (31.12)? Jaki Jest Stan Zachorowań w Woj. łódzkim? OGROM ZGONÓW! [RAPORT KORONAWIRUS]. https://lodz.se.pl/koronawirus-w-polsce-ile-zakazen-dzisiaj-31-12-jaki-jest-stan-zachorowan-w-woj-lodzkim-raport-koronawirus-aa-biAS-QgE2-yMyL.html.

[14] Urząd Statystyczny We Wrocławiu. https://wroclaw.stat.gov.pl.

[15] Urząd Statystyczny w Łodzi. https://lodz.stat.gov.pl.

[16] Raciborski F., Pinkas J., Jankowski M., Sierpiński R., Zgliczyński W.S., Szumowski Ł., Rakocy K., Wierzba W., Gujski M. (2020). Dynamics of the Coronavirus Disease 2019 Outbreak in Poland: An Epidemiological Analysis of the First 2 Months of the Epidemic. Pol. Arch. Intern. Med..

[17] Nowak B., Szymański P., Pańkowski I., Szarowska A., Życińska K., Rogowski W., Gil R., Furmanek M., Tatur J., Zaczyński A. (2020). Clinical Characteristics and Short-Term Outcomes of Patients with Coronavirus Disease 2019: A Retrospective Sin-Gle-Center Experience of a Designated Hospital in Poland. Pol. Arch. Intern. Med..

[18] Strategia Walki z Pandemią COVID-19. https://www.termedia.pl/mz/-Strategia-walki-z-pandemia-COVID-19-,39390.html.

[19] Dudink E., Essers B., Holvoet W., Weijs B., Luermans J., Ramanna H., Liem A., Opstal J., Dekker L., Dijk V. (2017). Acute Car-Dioversion vs a Wait-and-See Approach for Recent-Onset Symptomatic Atrial Fibrillation in the Emergency Department: Ra-Tionale and Design of the Randomized ACWAS Trial. Am. Heart J..

[20] D’Ascenzi F., Cameli M., Forni S., Gemmi F., Szasz C., Fabrizio V.D., Mechi M.T., Nocci M., Mondillo S., Valente S. (2021). Reduction of Emergency Calls and Hospitalizations for Cardiac Causes: Effects of Covid-19 Pandemic and Lockdown in Tuscany Region. Front. Cardiovasc. Med..

[21] Pluymaekers N.A.H.A., Dudink E.A.M.P., Luermans J.G.L.M., Meeder J.G., Lenderink T., Widdershoven J., Bucx J.J.J., Rienstra M., Kamp O., Van Opstal J.M. (2019). RACE 7 ACWAS Investigators. Early or Delayed Cardioversion in Recent-Onset Atrial Fibrillation. N. Engl. J. Med..

[22] Capucci A., Compagnucci P. (2020). Is Delayed Cardioversion the Better Approach in Recent-Onset Atrial Fibrillation? No. Intern. Emerg. Med..

[23] Wakai A., O’Neill J.O. (2003). Emergency Management of Atrial Fibrillation. Postgrad. Med. J..

